# Expression and Biochemical Characterization of a Novel Marine Chitosanase from *Streptomyces niveus* Suitable for Preparation of Chitobiose

**DOI:** 10.3390/md19060300

**Published:** 2021-05-24

**Authors:** Tong Chen, Gong Cheng, Siming Jiao, Lishi Ren, Chuanfang Zhao, Jinhua Wei, Juntian Han, Meishan Pei, Yuguang Du, Jian-Jun Li

**Affiliations:** 1 School of Chemistry and Chemical Engineering, University of Jinan, Jinan 250022, China; tongchen0319@163.com; 2 National Key Laboratory of Biochemical Engineering, National Engineering Research Center for Biotechnology (Beijing), Key Laboratory of Biopharmaceutical Production & Formulation Engineering, PLA, Institute of Process Engineering, Chinese Academy of Sciences, Beijing 100190, China; gcheng@ipe.ac.cn (G.C.); smjiao@ipe.ac.cn (S.J.); lsren@ipe.ac.cn (L.R.); jhwei@ipe.ac.cn (J.W.); hanjuntian@ipe.ac.cn (J.H.); 3 State Key Laboratory of Environmental Chemistry and Eco-Toxicology, Research Center for Eco-Environmental Sciences, Chinese Academy of Sciences, Beijing 100085, China; zhaochuanfang88@126.com

**Keywords:** *endo*-chitosanases, *Streptomyces niveus*, pH and temperature-rate profile, pH and thermal stability, chitobiose, partially acetylated chitooligosaccharide, structure modelling

## Abstract

It is known that bioactivities of chitooligosaccharide (COS) are closely related to the degree of polymerization (DP); therefore, it is essential to prepare COS with controllable DP, such as chitobiose showing high antioxidant and antihyperlipidemia activities. In this study, BLAST, sequence alignment and phylogenetic analysis of characterized glycoside hydrolase (GH) 46 *endo*-chitosanases revealed that a chitosanase Sn1-CSN from *Streptomyces niveus* was different from others. Sn1-CSN was overexpressed in *E. coli*, purified and characterized in detail. It showed the highest activity at pH 6.0 and exhibited superior stability between pH 4.0 and pH 11.0. Sn1-CSN displayed the highest activity at 50 °C and was fairly stable at ≤45 °C. Its apparent kinetic parameters against chitosan (DDA: degree of deacetylation, >94%) were determined, with *K*_m_ and *k_cat_* values of 1.8 mg/mL and 88.3 s^−1^, respectively. Cu^2+^ enhanced the activity of Sn1-CSN by 54.2%, whereas Fe^3+^ inhibited activity by 15.1%. Hydrolysis products of chitosan (DDA > 94%) by Sn1-CSN were mainly composed of chitobiose (87.3%), whereas partially acetylated chitosan with DDA 69% was mainly converted into partially acetylated COS with DP 2-13. This *endo*-chitosanase has great potential to be used for the preparation of chitobiose and partially acetylated COS with different DPs.

## 1. Introduction

Chitin is a β-1,4-linked polysaccharide of *N*-acetylglucosamine (GlcNAc, A) and is the second-largest natural organic, renewable resource. It is widely distributed in living organisms, including insects, arthropod shells, shrimps, crabs, and some higher plants and fungal cell walls [1]. Chitosan, which is the deacetylated product of chitin, is a linear polysaccharide, consisting of *N*-acetylglucosamine and glucosamine (GlcN, D) linked by β-1,4-glucosidic bonds [2], and possesses diverse biological activities such as antifungal activity, antibacterial activity, antiviral activity, etc. However, its application and commercial development are greatly restricted by its large molecular weight and poor water solubility [3]. By comparison, COS has a lower molecular weight and viscosity and better water solubility and, in some studies, exhibits better antifungal and antiviral activity and better ability to promote plant growth, etc. [4,5].

COS is the degradation product of chitosan and is the only natural oligosaccharide with a positive charge. COS can be prepared by chemical, physical, and enzymatic degradation [6]. Chemical methods mainly include oxidative and acidic degradation. However, these methods have the disadvantages such as violent reaction conditions, unwanted byproducts, poor selectivity, high monosaccharide content, uncontrollability, poor reproducibility, and serious environmental pollution [7]. Physical methods mainly involve photodegradation, ultrasonic and microwave degradation. Compared with chemical degradation, physical methods can be operated under mild conditions, with good controllability and simple operation, but they are difficult to obtain biologically active oligomers. Enzymatic approaches refer to enzymatic degradation of chitosan, which is advantageous over the other two approaches in mild reaction conditions, high yields, good selectivity, controllability and reproducibility, and beneficial to the environment. They have been widely used for COS production [6].

**Figure 1:**
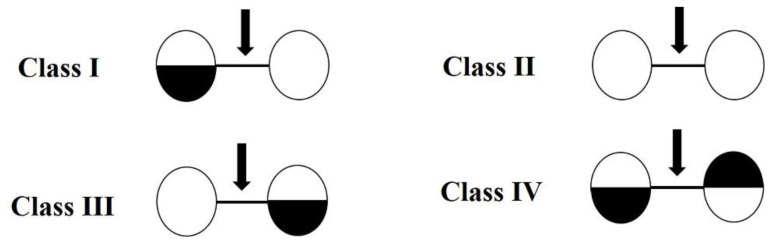
The classification of chitosanases according to cleavage specificity [18]. GlcN (D) is represented in white, and GlcNAc (A) is represented in black. For a position in which either A or D can be present, the circle is half-white and half-black. The cleavage site is represented by an arrow.

Chitosanases (EC 3.2.1.132) are a class of enzymes that hydrolyze *endo*-β-1,4-glycosidic linkages in chitosan, leading to the formation of COS with different DPs [8]. They are widely distributed in bacteria, fungi, actinomyces, viruses and are some plant tissues [8,9]. According to the Carbohydrate Active Enzyme Database (CAZy, http://www.cazy.org/, accessed on 8 February 2021), the identified *endo*-chitosanases are divided into six glycoside hydrolase (GH) families: GH 5, GH 7, GH 8, GH 46, GH 75, and GH 80. The well-characterized *endo*-chitosanases are the members of GH 8, GH 46, GH 75, and GH 80 [10,11,12]. GH 46, GH 75, and GH 80 contain exclusively chitosanases. Those from the GH 46 have a strong catalytic ability [13] and are mainly from various bacteria, including *Bacillus subtilis*, *Kitasatospora setae*, *Pseudomonas* sp., and *Streptomyces* sp. [13,14,15,16], while fungal chitosanases are mainly distributed in GH 75. In addition, according to the specific cleavage sites of chitosanases, chitosanases are classified into four categories (Figure 1). Class I chitosanases, such as the *Streptomyces* sp. N174 chitosanase (belonging to GH 46), can cleave GlcNAc-GlcN and GlcN-GlcN; Class II chitosanases, such as the *Bacillus* sp. No. 7-M chitosanase (belonging to GH 8) can only cleave GlcN-GlcN; Class III chitosanases, for example, the *Bacillus circulans* MH-K1 chitosanase (belonging to GH 46) can cleave both GlcN-GlcN and GlcN-GlcNAc; Class IV chitosanases, for instance, the ones from *Pseudomonas* sp. A-01 and *Amycolatopsis* sp. CsO-2 (belonging to GH 46 family) can cleave all three bonds [8,16]. Strikingly, the chitosanase from *Streptomyces coelicolor* A3(2) can cleave all bonds, even including GlcNAc- GlcNAc, though it prefers GlcN-units. For this type of specificity, no class was assigned. Given the observation that chitosanases’ abilities to cleave bonds at GlcNAc residues positioned at subsite (−1) or (+1), Weikert et al. suggested a new chitosanase classification system, which is based on specificities and preferences of subsites (−2) to (+2) towards substrates with different fractions of acetylation (FA) [17,18].

The bioactivities of COS depend closely on its structure and physicochemical properties. The DP, degree of deacetylation (DDA), charge distribution, and the oligomer structure pattern have an important influence on its bioactivities. In particular, the DP of COS is a very important factor in the study of structure–function relationship of COS. Chen et al. reported that the oral bioavailability of chitobiose was higher than that of chitotriose at all doses (30, 100, and 300 mg/kg) examined [18]. In addition, it was found that chitobiose effectively protected rats from hepatic injury induced by carbon tetrachloride and showed the most active antihyperlipidemia effect [19,20]. Moreover, it has been reported that the antibacterial activity required COS with a DP of at least 5, and the inhibitory effects improved with increasing DPs of COS [21]. Furthermore, chitohexaose and chitoheptaose exhibited the most effective activities in alleviating chilling stress to wheat seedlings [22]. COS with DDA 50% was the most effective in alleviating salt stress to wheat seedlings, indicating that the activity of COS was closely related to its DDAs too [23].

**Figure 2:**
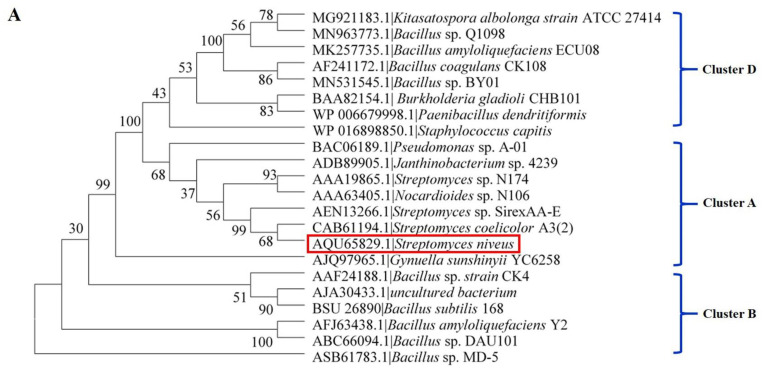

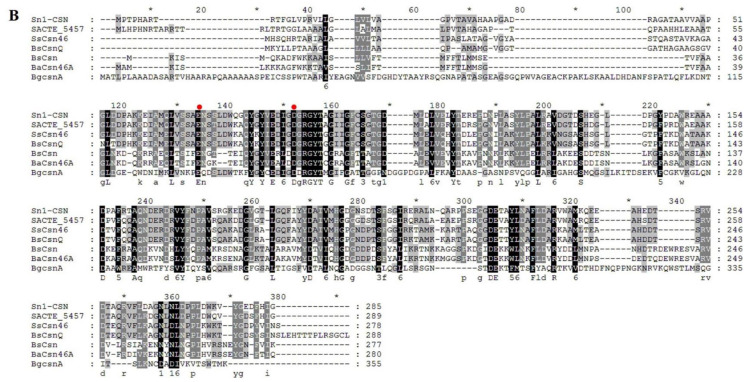
Phylogenetic, sequence analysis, and classification of GH46 chitosanases [9]. (**A**) Phylogenetic analysis and classification of GH46 chitosanases. The maximum likelihood method was used to construct the phylogenetic tree. The homologous sequences were found using BLAST at NCBI. Sequences included in the alignment were derived from *Bacillus amyloliquefaciens* YX01 (BaCsn46A, GenBank No. AFJ63438.1) [14], *Bacillus* sp. MD-5 (Csn-BAC, GenBank No. CP021911.1) [3], *Streptomyces albolongus* ATCC27414 (Csn21c, GenBank No. MG921183.1) [27], *Streptomyces* sp. N174 (SsCsn46, GenBank No. AAA19865.1) [16], *Bacillus amyloliquefaciens* ECU08 (BaCSN46B, GenBank No. MK257735.1) [28], *Bacillus subtilis* 168 (BsCsn, GenBank No. BSU_26890) [25], *Staphylococcus capitis* (Csn-CAP, GenBank No. WP_016898850.1) [29], *Bacillus coagulans* CK108(TCH-2, GenBank No. AAL40906.1) [30], *Bacillus* sp. DAU101(CSN-SP, GenBank No. ABC66094.1) [31], *Bacillus* sp. strain CK4 (choK, GenBank No. AAF24188.1) [32], *Burkholderia gladioli* CHB101(BgcsnA, GenBank No. BAA82154.1) [33], *Nocardioides* sp. N106 (csnN106, GenBank No. AAA63405.1) [34], *Pseudomonas* sp. A-01 (Cto1, GenBank No. BAC06189.1) [35], *Streptomyces coelicolor* A3(2) (ScCsnA, GenBank No. CAB61194.1) [36], uncultured bacterium(CsnA, GenBank No. AJA30433.1) [37], *Gynuella sunshinyii* YC6258 (GsCsn46A, GenBank No. AJQ97965.1) [10], *Paenibacillus dendritiformis* (Csn-PD, GenBank No. WP_006679998.1) [38], *Bacillus* sp. BY01 (BsCsnB, GenBank No. MN531545.1) [39], *Bacillus* sp. Q1098(BsCsnQ, GenBank No. MN963773.1) [40], *Janthinobacterium* sp. 4239(Cho4239-1, GenBank No. ADB89905.1) [41], and *Streptomyces* sp. SirexAA-E (SACTE_5457, GenBank No. AEN13266.1) [24]. (**B**) Multiple sequence alignment of some GH 46 chitosanases. Putative conserved residues involved in catalysis were marked with red dots above.

Therefore, the identification and development of new chitosanases to produce COS with controllable DP and DDA are essential. Until now, few marine chitosanases have been characterized in detail and used for COS production [15]. In this study, one new GH 46 chitosanase Sn1-CSN (GenBank: AQU65829.1) from marine microorganism *Streptomyces niveus* was found by subjecting a well-characterized chitosanase from *Streptomyces* sp. N174 (CSN-174) to a BLAST search (Figure 2A), and it showed the highest sequence identity (75.6%) to the reported SACTE_5457 chitosanase from *Streptomyces* sp. SirexAA-E, implying that Sn1-CSN might have different enzymological properties from those characterized GH 46 chitosanases [24]. It was overexpressed and purified, and its biological properties, kinetic parameters, and degradation products of chitosan (DDA > 94%) and partially acetylated chitosan (DDA 69%) were investigated. In addition, structure modelling of Sn1-CSN with docked chitobiose and chitotriose was performed.

## 2. Results and Discussion

### 2.1. Sequence Alignment and Phylogenetic Analysis of Sn1-CSN with Characterized GH 46 Chitosanases

Though many GH 46 chitosanases have been identified and characterized, until now, few marine GH 46 chitosanases have been investigated [15,25]. One GH 46 chitosanase (GenBank: AQU65829.1) from *Streptomyces niveus* in deep-sea sediment was found by subjecting the chitosanase CSN-174 from *Streptomyces* sp. N174 (GenBank: AAA19865.1) to a BLAST search. The chitosanase from *Streptomyces niveus* was named Sn1-CSN in the current study.

The GH 46 family chitosanases can be further grouped into five subclasses: Cluster A–E, among which Cluster C chitosanases have been found exclusively in viruses, whereas Cluster E groups together proteins for which no enzymatic activity has been reported so far [9]. Therefore, these two subclasses were not included for multiple sequence alignment and phylogenetic analysis (Figure 2). Sn1-CSN exhibited the highest sequence identity (75.6%) to SACTE_5457 (GenBank AEN13266.1) from *Streptomyces* sp. SirexAA-E, suggesting that Sn1-CSN is different from those characterized GH 46 chitosanases at least in amino acid sequence (Figure S1, Supplementary Materials) [24]. Sn1-CSN shares the same branch with SACTE_5457 and ScCsnA from *Streptomyces coelicolor* A3(2) (GenBank No. CAB61194.1) and belongs to Cluster A (Figure 2A). Two conserved glutamic acid (Glu40) and aspartic acid (Asp58) residues were considered as the catalytic residues in Sn1-CSN (Figure 2B) [24]. SACTE_5457 could not degrade chitin but can hydrolyze chitosans having different DDAs (75–>90%) with markedly different *k_cat_/K_M_* values [24]. Based on the above analysis, it was presumed that Sn1-CSN might have some different enzymological properties from those reported GH 46 chitosanases. Therefore, Sn1-CSN was chosen for further investigation.

The modeled structure of Sn1-CSN based on homologous enzymes (PDB ID: 4ILY from *Streptomyces* sp. SirexAA-E; PDB ID: 1CHK from *Streptomyces* N174) was obtained by the I-TASSER server. Five top-ranking 3D models were generated. Each model was validated based on confidence score (C-score), template modeling score (TM-score), the root-mean-square deviation (RMSD), and cluster density. In general, models with C-score > −1.5 have a correct fold. Model 1 had the highest C-scorevalue (1.31), reflecting a model of better quality (TM-score = 0.90 ± 0.06 and RMSD = 3.2 ± 2.3 Å) (Figure S1, Supplementary Materials). Similar to homologous GH 46 chitosanases, the modeled three-dimensional structure of Sn1-CSN was composed of two α-helical domains connected by a bent α-helix (residues 126–151). The smaller one of the two domains, designated here as the N-terminal domain, is composed of residues 46–123, while the larger domain, named as the C-terminal domain, includes residues 1–45 and 152–255 [9,16].

**Figure 3:**
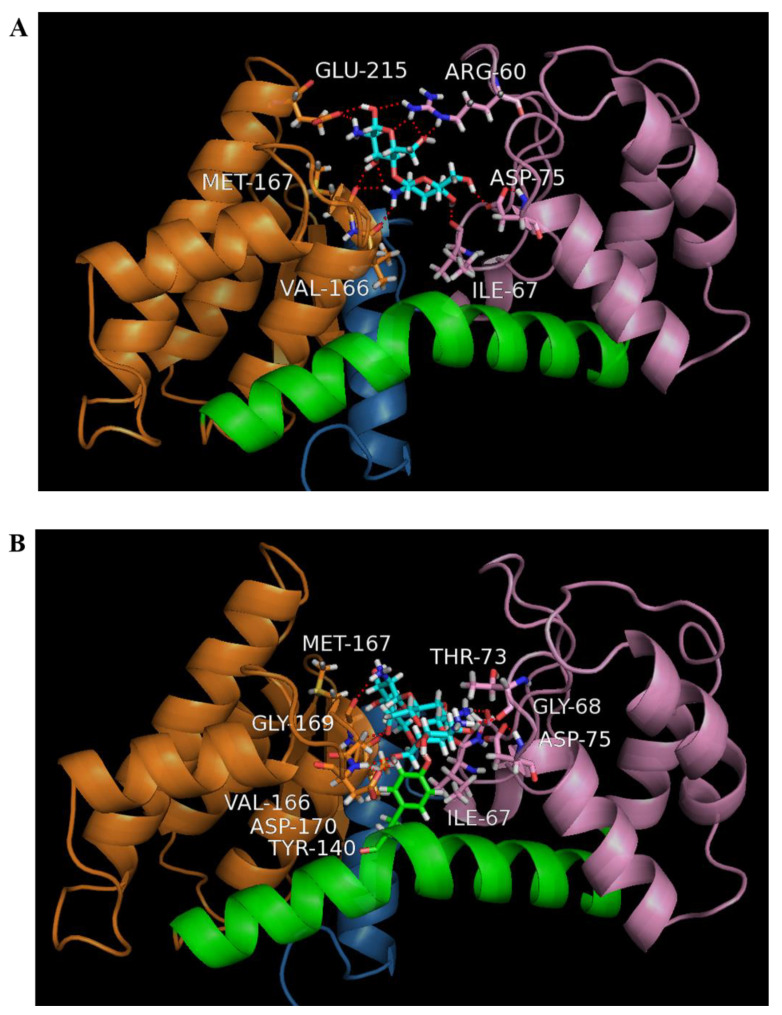
Molecular docking of Sn1-CSN and chitobiose (**A**) and chitotriose (**B**). The modeled structure of Sn1-CSN was displayed in the ribbon mode, and the ligands, chitobiose and chitotriose, and amino acids participating in hydrogen bonding were shown as a ball-and-stick model. Residues 1–45, 46–123, 152–255 corresponding to domains were colored blue, pink, and orange, respectively, while the bent α-helix consisting of residues 124–151 was highlighted in green.

In order to insight into the possible interaction mode of Sn1-CSN with chitosan, its binding interactions with chitobiose and chitotriose were done by molecular docking. The TotalScores of binding of Sn1-CSN with chitobiose and chitotriose were 6.08 and 7.12, respectively, indicating that the binding interaction of Sn1-CSN with chitobiose is weaker than that with chitotriose. Structural analyses showed that chitobiose binds with Sn1-CSN by electrostatic interaction with Glu215 and hydrogen bonding with Arg60, Ile67, Asp75, Val166, and Met167, whereas chitotriose interacts with Sn1-CSN by electrostatic interaction with Asp75 and hydrogen bonding with Ile67, Gly68, Thr73, Asp75, Tyr140, Val166, Met167, Gly169, and Asp170 (Figure 3). Therefore, more hydrogen bonds are involved in the interaction of Sn1-CSN with chitotriose, leading to stronger binding interaction between Sn1-CSN and chitotriose than that between Sn1-CSN and chitobiose. Some involved residues are highly conserved among GH 46 chitosanases [9,26].

### 2.2. Overexpression and Purification of Sn1-CSN in E. coli

The gene encoding Sn1-CSN was cloned into pET-22b and overexpressed in *E. coli* BL21(DE3). The overexpressed protein was purified by nickel-chelating affinity chromatography (the predicted molecular weight based on the amino acid sequence: 29.8 kDa) (Figure S2, Supplementary Materials). The yield of Sn1-CSN was about 30 mg per liter LB medium.

### 2.3. Determination of pH Optima and pH Stability of Sn1-CSN

**Figure 4:**
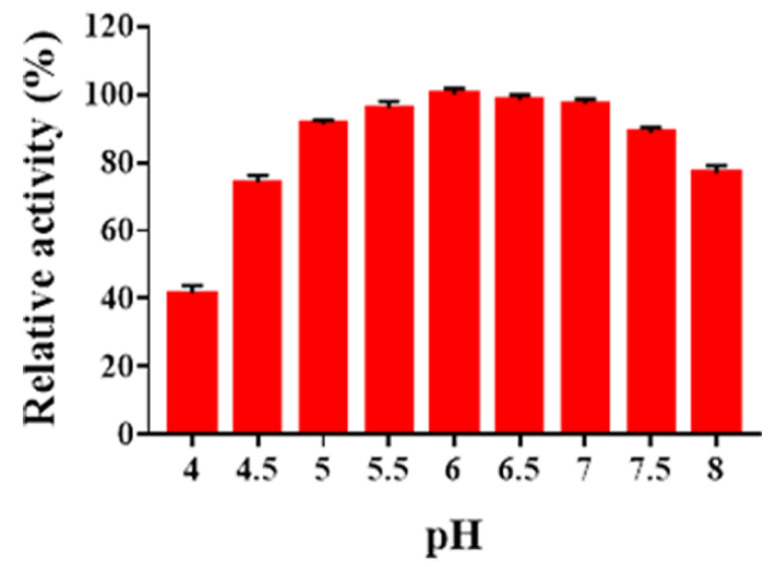
The effects of pH on Sn1-CSN activity. The percentages of activities at different pH values against the one at optimal pH (6.0) and 37 °C were calculated. The mean values ± standard deviations of three replicates were shown.

As shown in Figure 4, no obvious activities were detected below pH 4.0 and above pH 9.0 (data not shown). Sn1-CSN showed the highest activity at pH 6.0 and retained residual activity above 80% between pH 5.0 and pH 7.5. It maintained 74.1% and 77% of residual activity at pH 4.5 and pH 8.0, respectively.

In comparison with some characterized GH 46 chitosanases (Table S1, Supplementary Materials), as Sn1-CSN most of them exhibited the highest activity below pH 7.0, which were classified as acidic chitosanases, including BaCsn46A from *Bacillus amyloliquefaciens* YX01 (pH 6.0) [14], SsCsn46 from *Streptomyces* sp. N174 (pH 5.5) [16], BaCSN46B from *Bacillus amyloliquefaciens* ECU08 (pH 6.5) [28], BsCsn form *Bacillus subtilis* 168 (pH 5.0–6.0) [25], TCH-2 from *Bacillus coagulans* CK108 (pH 6.5) [30], BgcsnA from *Burkholderia gladioli* CHB101(pH 5.6) [33], Cto1 from *Pseudomonas* sp. A-01(pH 5.0) [35], ScCsnA from *Streptomyces coelicolor* A3(2) (pH 4.1) [36], CsnA from uncultured bacterium (pH 6.0) [37], GsCsn46A from *Gynuella sunshinyii* YC6258(pH 5.5) [10], BsCsnB from *Bacillus* sp. BY01(pH 5.0) [39], BsCsnQ from *Bacillus* sp. Q1098(pH 5.3) [40], Cho4239-1 from *Janthinobacterium* sp. 4239 (pH 5.0) [41], and SACTE_5457 from *Streptomyces* sp. SirexAA-E (pH 6.0) [24]. Csn-BAC from *Bacillus* sp. MD-5 (pH 7.0) [3], Csn-CAP from *Staphylococcus capitis* (pH 7.0) [29], Csn-PD from *Paenibacillus dendritiformis* (pH 7.0) [38], CSN-SP from *Bacillus* sp. DAU101 (pH 7.5) [31], choK from *Bacillus* sp. strain CK4 (pH 7.5) [32], and Csn21c from *Streptomyces albolongus* ATCC27414 (pH 8.0) [27] were most active at pH ≥ 7.0. Thus, it seems that most of GH 46 chitosanases belong to acidic ones.

**Figure 5:**
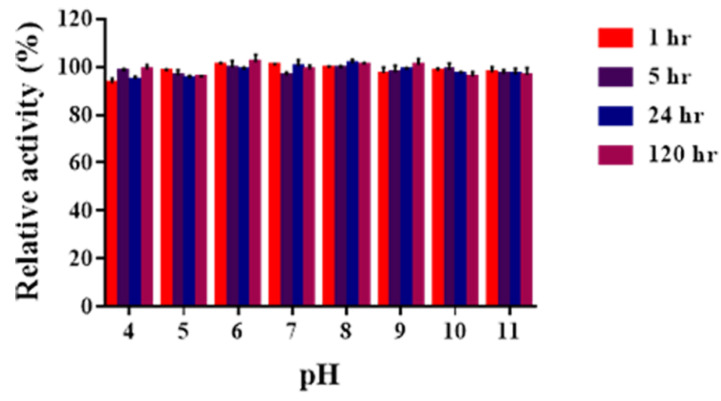
pH stability of Sn1-CSN. The pH stability assay was investigated by first preincubating Sn1-CSN in Britton-Robinson buffer at different pH values (pH 4.0–11.0) at 4 °C for 1 h, 5 h, 24 h, and 120 h, respectively. The residual activities were then measured immediately under standard conditions. The initial activity at optimal pH (6.0) was taken as 100%, and the percentages of the residual activities at different time points and pH values against the one at optimal pH (6.0) and 37 °C were calculated. The mean values ± standard deviations of three replicates were shown.

The pH stability of Sn1-CSN was investigated after being preincubated for a fixed time over the pH range of 4.0–11.0 (Figure 5). Sn1-CSN was stable over the pH range of 4.0–11.0, retaining > 90.0% original activity after 120 h. Therefore, Sn1-CSN was very stable between pH 4.0 and pH 11.0 and exhibited superior pH stability over a wide pH range.

Similar to Sn1-CSN, some GH46 chitosanases were also stable over a wider pH range (Table S1, Supplementary Materials), for example, BaCsn46A(pH 5.0–9.5) [14], Csn21c (pH 6.0–10.0) [17], BaCSN46B(pH 3.0–10.5) [28], GsCsn46A(pH 4.0–9.0) [10], BsCsn (pH 2.0–9.0) [25], BsCsnB(pH 3.0–7.5) [29], and (pH 3.6–9.8)BsCsnQ [40]. By contrast, some GH46 chitosanases were only stable over a narrow pH range (Table S1, Supplementary Materials), including Csn-BAC(pH 5.5–8.5) [3], SsCsn46(pH 5.0–6.5) [16], Csn-CAP (pH 5.0–7.0) [29], CsnA (pH 4.5–6.5) [37], Csn-PD (pH 6.0–7.0) [38] and Cto1 (pH 5.0–8.0) [35]. It seems that SsCsn46(pH 5.0–6.5) [16], Csn-CAP (pH 5.0–7.0) [29], CsnA(pH 4.5–6.5) [37], and Csn-PD (pH 6.0–7.0) [38] were only stable under acidic conditions (Table S1, Supplementary Materials). Therefore, GH46 chitosanases exhibit markedly different pH stability.

### 2.4. Determination of Temperature Optima and Thermal Stability of Sn1-CSN

**Figure 6:**
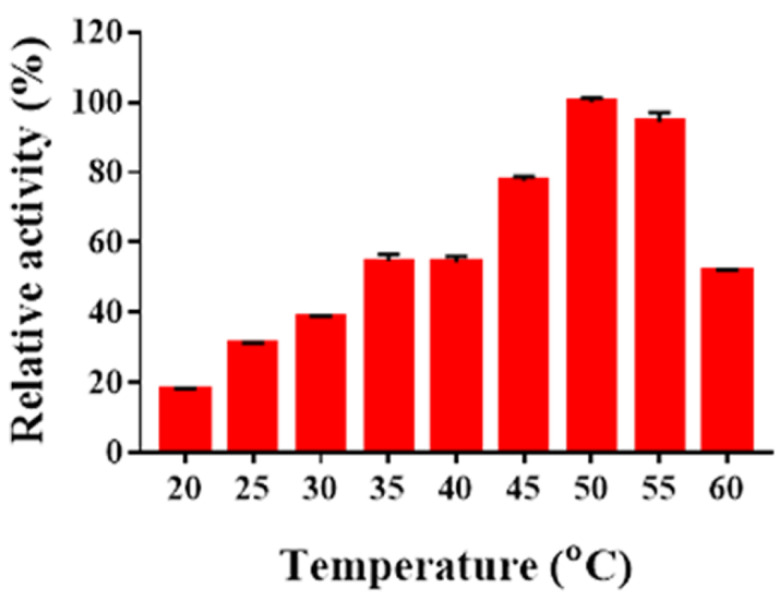
The effects of temperature on Sn1-CSN activity. The percentages of activities at different temperatures against the one at optimal temperature (50 °C) and pH 6.0 were calculated. The mean values ± standard deviations of three replicates were shown.

The optimal temperature of Sn1-CSN was determined (Figure 6). It showed the highest activity at 50 °C and kept >70% of residual activity between 45 °C and 55 °C and retained less than 55% of residual activity at 60 °C and below 40 °C.

Some GH46 chitosanases, such as BaCsn46A [14], Csn21c [27], CSN-SP [31], and ScCsnA [36], also showed the highest activity at 50 °C (Table S1, Supplementary Materials). Csn-BAC(40 °C) [3], Csn-CAP (30 °C) [29], GsCsn46A (30 °C) [10], Csn-PD (45 °C) [38], BsCsnB (35 °C) [39], and Cho4239-1(45 °C) [41] were most active below 50 °C, whereas SsCsn46 (65 °C) [16], TCH-2 (65 °C) [30], and BsCsnQ (60 °C) [40] exhibited maximum activity above 55 °C (Table S1, Supplementary Materials). GH46 chitosanases show greatly different optimal temperatures ranging from 30 °C to 65 °C.

**Figure 7:**
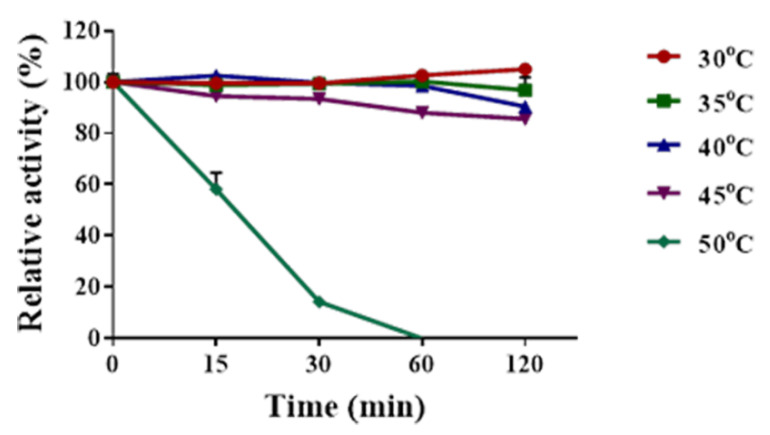
Thermal stability of Sn1-CSN. Sn1-CSN was pre-incubated for varied times (15 min to 2 h) at pH 6.0, and 30 °C, 35 °C, 40 °C, 45 °C, and 50 °C, respectively, and samples were chilled on ice for at least 15 min. Afterwards, the residual activities were measured under standard conditions. The original activity at pH 6.0 and 37 °C was taken as 100%, and the percentage of the residual activity at different time points and temperatures against the initial one was calculated. The mean values ± standard deviations of three replicates were shown.

The thermal stability of Sn1-CSN was investigated after being preincubated for a fixed period at pH 6.0, and 30 °C, 35 °C, 40 °C, 45 °C, and 50 °C, respectively (Figure 7). Sn1-CSN was very stable below 35 °C and lost 9.6–14.4% of original activity after incubated at 40 °C and 45 °C for 2 h. It was completely inactivated at 50 °C after 1 h. Therefore, Sn1-CSN was a thermolabile chitosanase and was fairly stable below 45 °C.

Similar to Sn1-CSN, some GH46 chitosanases, including SsCsn46 (<45 °C) [16], BsCsn (<45 °C) [25], GsCsn46A (<40 °C) [10], BsCsnB (<30 °C) [39], and BsCsnQ (<30 °C) [40], were only stable at <45 °C (Table S1, Supplementary Materials). By comparison, BaCsn46A(<50 °C) [14], Csn-BAC (<50 °C) [3], Csn21c (<65 °C) [27], BaCSN46B (<55 °C) [28], Csn-CAP (<50 °C) [29], CsnA (<55 °C) [37], Csn-PD (<50 °C) [38], and Cho4239-1(<50 °C) [41] were stable below 65 °C, while TCH-2 (<80 °C) [30] and choK (<80 °C) [32] belonged to thermophilic GH46 chitosanases (Table S1, Supplementary Materials). Thermal stability of GH46 chitosanases varies widely from <30 °C to <80 °C.

### 2.5. Determination of Kinetic Parameters of Sn1-CSN

**Figure 8:**
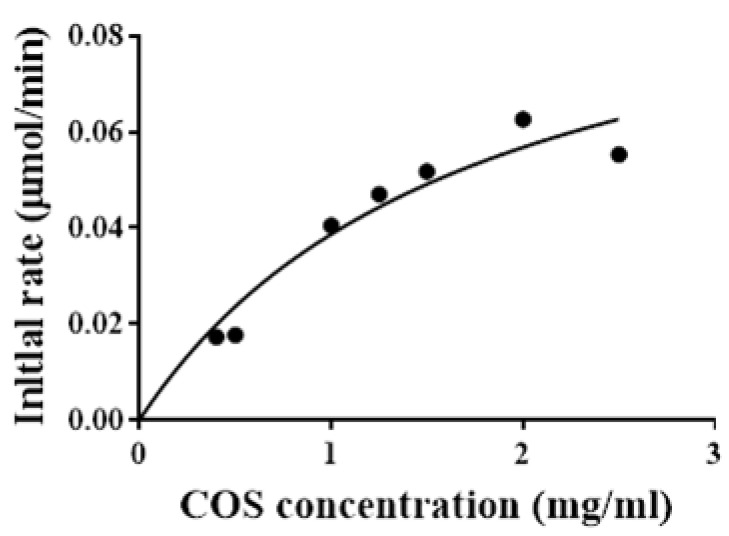
The effects of the chitosan concentration on Sn1-CSN activity. The mean values ± standard deviations of three replicates for each substrate concentration were shown.

The kinetic parameters of recombinant Sn1-CSN against chitosan were determined by substrate hydrolysis from 0.4 to 2.5% (*w*/*v*) for 15 min. The deduced kinetic values were apparent parameters since saturation was not achieved even when high chitosan (DDA > 94%) concentrations were used (Figure 8). The apparent *K_m_*, *k_cat_*, and *V_max_* values of Sn1-CSN for chitosan were 1.8 ± 0.3 mg/mL, 88.3 ± 7.6 s^−1^, and 1800 ± 145 μmol/min/mg, respectively.

In comparison with the kinetic parameters of other GH 46 chitosanases (Table S1, Supplementary Materials), the apparent *K_m_* value of Sn1-CSN is much lower than those of Csn21c (7.4 mg/mL) [27] and CsnA (7.29 mg/mL) [37], showing higher binding affinity towards chitosan than Csn21c and CsnA. However, its apparent *K_m_* value is significantly higher than those of SsCsn46 (0.029 mg/mL) [16] and ScCsnA (0.054 mg/mL) [36], demonstrating lowering binding affinity towards chitosan than SsCsn46 and ScCsnA. The *K_m_* values of other GH46 chitosanases, such as BaCsn46A (2.8 mg/mL) [14], BsCsn (1.57 mg/mL) [25], choK (0.8 mg/mL) [32], GsCsn46A (1.97 mg/mL) [10], and SACTE_5457 (2.2 mg/mL) [24], were close to that of Sn1-CSN. Comparison of apparent *V_max_* value of Sn1-CSN with other GH46 chitosanases (Table S1, Supplementary Materials), BsCsn (31,800 μmol/min/mg) [25] and BaCsn46A(7142.9 μmol/min/mg) [14] showed significantly higher apparent *V_max_* values than Sn1-CSN, whereas it exhibited comparable apparent *V_max_* value with that of CsnA (2373 μmol/min/mg) [37] and much higher apparent *V_max_* than those of Csn21c(263.1 μmol/min/mg) [27], SsCsn46 (27 μmol/min/mg) [16], TCH-2 (516 μmol/min/mg) [30], choK (173 μmol/min/mg) [32], ScCsnA (37 μmol/min/mg) [36], GsCsn46A (385.6 μmol/min/mg) [10], and SACTE_5457 (25 μmol/min/mg) [24]. Kinetic parameters of GH 46 chitosanases from different microorganisms vary greatly.

### 2.6. Effects of Metal Ions on Enzyme Activity

**Figure 9:**
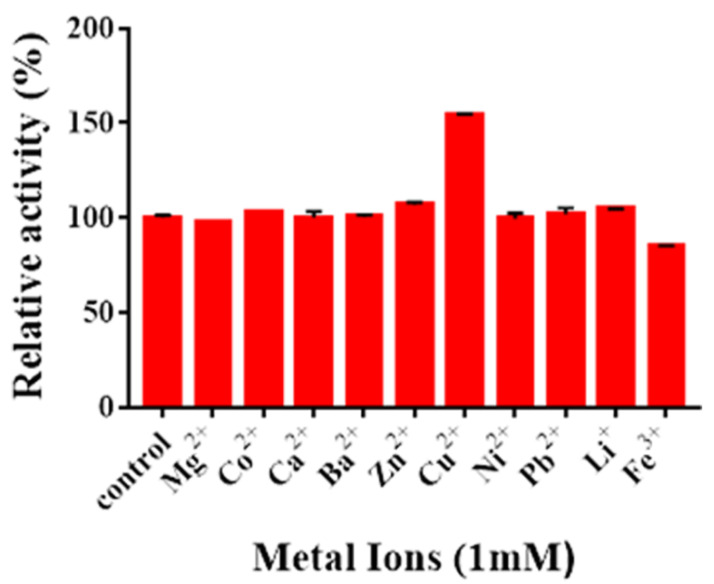
The effects of metal ions on Sn1-CSN activity. The effects of metal ions on the catalytic activity of Sn1-CSN were determined by adding 1 mM of various metal ions to the standard enzyme assay system (pH 6.0 and 37 °C for 15 min). The activity at pH 6.0 and 37 °C in the absence of metal ions (control) was taken as 100%, and the percentage of the activity in the presence of different metal ions against the control was calculated. The mean values ± standard deviations of three replicates were shown.

The effects of metal ions on Sn1-CSN activity were examined, too (Figure 9). Cu^2+^ upregulated the activity of Sn1-CSN by 54.2%, while Fe^3+^ decreased the activity of Sn1-CSN by 15.1%. Other metal ions did not show obvious influence on the catalytic activity of Sn1-CSN.

Combining our results with the published ones, it seems that different metal ions showed different effects against characterized GH46 chitosanases from different microorganisms, even same metal ions exhibited completely opposite effects (Table S2, Supplemental files). For example, Cu^2+^ upregulated the activities of Csn-BAC [3], Sn1-CSN, Csn-CAP [29], BsCsnB [39], BsCsnQ [40], and Csn21c [27], but it reduced activities of Cto1 [35], CsnA [37], and CSN-SP [31]. Ni^2+^ inhibited activities of CsnA [37], CSN-SP [31], Csn-BAC [3], Cto1 [35], and Csn-CAP [29], whereas it showed no impact on Sn1-CSN. Ba^2+^ interfered with activities of CsnA [37], CSN-SP [31], Cto1 [35], and Csn-CAP [29], while it exhibited no influence on Sn1-CSN, Csn-BAC [3], and Csn21c [27]. However, it improved activities of BsCsnB [39] and BsCsnQ [40]. Ca^2+^ activated CSN-SP [31], BsCsnQ [40], BsCsnB [39], and Csn-BAC [3], whereas it acted as an inhibitor for CsnA [37], Csn-CAP [29], Cto1 [35], and Csn21c [27] and showed no influence on Sn1-CSN. Although Pb^2+^ did not affect activity of Sn1-CSN at all, it significantly inhibited CsnA [37]. Zn^2+^ inhibited CsnA [37], CSN-SP [31], Csn21c [27], Cto1 [35], and Csn-BAC [3], while it enhanced activities of BsCsnQ [40], Csn-CAP [29], and Sn1-CSN. Co^2+^ activated Csn-BAC [3], BsCsnQ [40], and Csn-CAP [29], but it interfered with Cto1 [35], CsnA [37], CSN-SP [31], and BsCsnB [39] and did not influence Sn1-CSN and Csn21c [27]. Mg^2+^ acted as an activator for BsCsnB [39], BsCsnQ [40], and CSN-SP [31], whereas it was an inhibitor for CsnA [37], Csn21c [27], and Csn-CAP [29] and showed no effects on Sn1-CSN, Csn-BAC [3], and Cto1 [35]. Fe^3+^ inhibited CsnA [37], BsCsnB [39], Csn21c [27], BsCsnQ [40], and Cto1 [35]. Li^+^ improved activities of BsCsnB [39], CSN-SP [31], and BsCsnQ [40]; however, it acted as an inhibitor for CsnA and did not influence activity of Cto1. It is worth pointing out that the underlying mechanism(s) of different impact of metal ions on GH 46 chitosanases from different microbial resources need(s) further investigation.

### 2.7. Identification of Hydrolysis Products of Chitosan Catalyzed by Sn1-CSN

**Figure 10:**
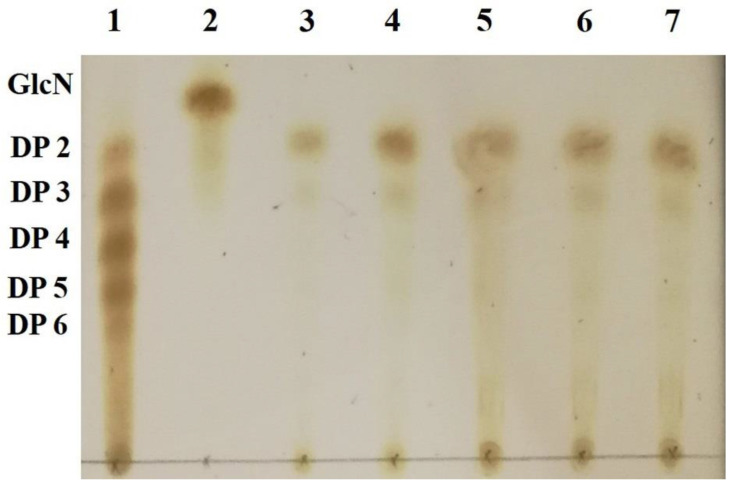
Thin-layer chromatography (TLC) analysis of hydrolytic products of chitosan catalyzed by Sn1-CSN over time. TLC conditions: butan-1-ol: acetic acid: water = 9:4:7) (*v*/*v*/*v*). Lane 1, standard chitosan oligomers (DP 2-6); Lane 2, glucosamine; Lanes 3–7, enzymatic hydrolysates of chitosan after 0.5 h, 1 h, 2 h, 4 h, and 24 h.

**Figure 11:**
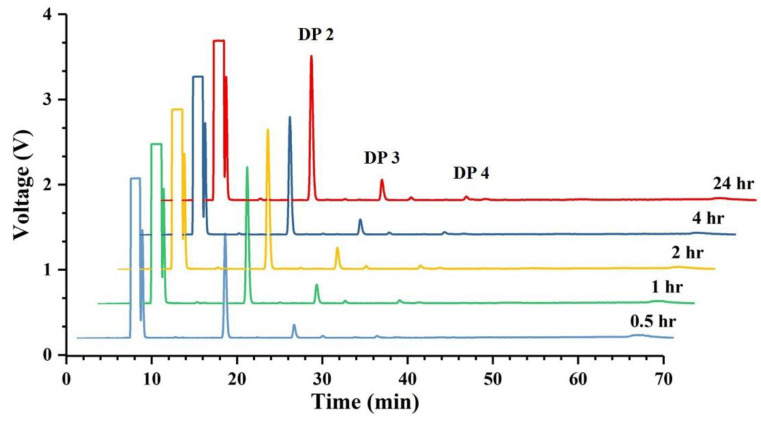
High-performance liquid chromatography (HPLC) analysis of hydrolytic products of chitosan catalyzed by Sn1-CSN over time.

**Figure 12:**
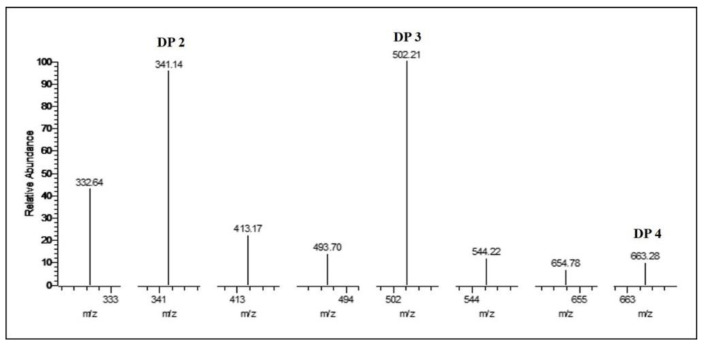
MS analysis of hydrolytic products of chitosan catalyzed by Sn1-CSN after 4 h.

Thin-layer chromatography (TLC) analysis of soluble sugars released from chitosan (DDA > 94%) by Sn1-CSN indicated that chitobiose was predominantly produced at the stage of initial 30 min and accumulated as hydrolysis continued, and less chitotriose was also generated (Figure 10). No monomer glucosamine (GlcN, D) was observed even after 24 h, suggesting that it was an *endo*-acting enzyme. The HPLC results further confirmed the above results, with chitobiose (DP2), chitotriose (DP3), and chitotetraose (DP4) accounting for 87.3%, 11.3%, and 1.4%, respectively (hydrolysis time of 4 h) (Figure 11). The formation of chitobiose, chitotriose, and chitotetraose were also proved by MS (Figure 12).

Compared with degradation products of other GH46 chitosanases (Table S1, Supplementary Materials), most of them produced COS with DP 2,3, including Csn-BAC [3], BaCSN46B [28], Csn-CAP [29], BsCsnB [39], BsCsnQ [40], Cho4239-1 [41], SACTE_5457 [24], and csnN106 [34], while hydrolysis products of CSN-SP [31] and GsCsn46A [10] had similar DP to 2-4. CsnA [37], choK [32] and BsCsn [25] hydrolyzed chitosan into COS with DP 2-6, whereas BaCsn46A(DP 2-10) [14], Csn21c(DP 1,2) [27], SsCsn46 (DP 3-5) [16], TCH-2 (DP 1-6) [30], and Csn-PD (DP 2) [38] produced COS with different DPs. It seems that most of GH46 chitosanases have a tendency to produce COS with lower DPs such as 2,3.

### 2.8. Analysis of Hydrolysis Products of Partially Acetylated Chitosan Catalyzed by Sn1-CSN

**Figure 13:**
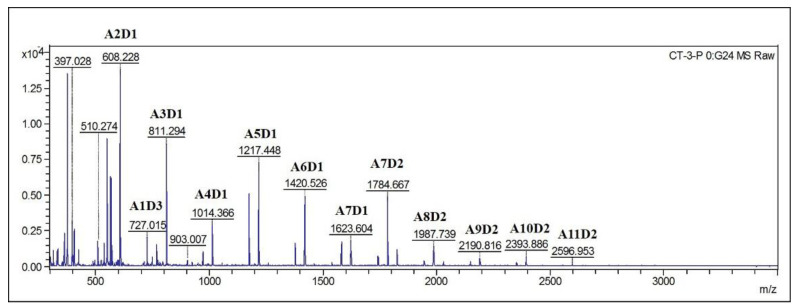
MS analysis of hydrolysis products of partially acetylated catalyzed chitosan by Sn1-CSN after 4 h. A (*N*-acetylglucosamine), D (glucosamine).

Since it has been found degree and position of acetylation of COS had important effects on biological activities of COS, partially acetylated chitooligosaccharides were also prepared from partially acetylated chitosan by Sn1-CSN. TLC results of partially acetylated chitosan (DDA 69%) were very similar to those of chitosan (DDA > 94%): chitobiose, chitotriose, and chitotetraose were detected, and chitobiose was still the main degradation product (Figure S3, Supplementary Materials), so were the HPLC results (Data not shown). However, MALDI-TOF MS analysis indicated that COSs with different DPs (DP 2–13) and different compositions of *N*-acetylglucosamine and glucosamine were clearly observed (Figure 13). Therefore, Sn1-CSN can be used to produce partially acetylated COS (paCOS).

It has been reported that paCOS also possess numerous bioactivities and have already been implemented in different applications [42]. However, few chitosanases have been used to produce them [43,44]. Therefore, Sn1-CSN might become a useful tool enzyme for the production of paCOS.

## 3. Materials and Methods

### 3.1. Materials

Unless specified, all chemicals were of analytical grade and purchased from Sigma (St. Louis, MO, USA), Aladdin (Shanghai, China), or Beijing Solarbio Science & Technology Co. Ltd. (Beijing, China). The codon-optimized gene encoding chitosanase from *Streptomyces niveus* (Sn1-CSN, GenBank No. AQU65829.1) was synthesized by Beijing Genomics Institute (China) (the codon-optimized gene sequence in Supplementary Materials). All restriction endonucleases were from Thermo Fisher Scientific (Waltham, MA, USA) or Takara Biotechnology (Otsu, Shiga, Japan). The kits used for molecular cloning were from Omega Bio-tek (Norcross, GA, USA) or Takara Biotechnology (Otsu, Shiga, Japan). Nickel column and the expression vector pET-22b (+) were from Novagen (Frankfurter Straße, Darmstadt, Germany).

### 3.2. Bacterial Strains, Plasmids, and Media

*E. coli* DH5α was used for routine DNA transformation and plasmid isolation. *E. coli* BL21(DE3) was utilized for chitosanase overexpression. *E. coli* strains were routinely grown in Luria–Bertani broth at 37 °C with aeration or on LB supplemented with 1.5% (*w*/*v*) agar. Ampicillin (100 μg/mL) was added when required.

### 3.3. Sequence Analysis and Phylogenetic Tree Analysis

The homologous sequences were searched using the BLAST online server (http://blast.ncbi.nlm.nih.gov/Blast.cgi, accessed on 10 January 2020). Multiple sequence alignment was performed by Clustal Omega (https://www.ebi.ac.uk/Tools/msa/clustalo/, accessed on 19 May 2021). The maximum likelihood phylogenetic tree was constructed from multiple alignment of Sn1-CSN and characterized GH 46 chitosanases through MEGA6. For multiple sequence alignment and phylogenetic analysis, sequences included were derived from some characterized GH46 chitosanases, including *Bacillus amyloliquefaciens* YX01 (BaCsn46A, GenBank No. AFJ63438.1) [14], *Bacillus* sp. MD-5 (Csn-BAC, GenBank No. CP021911.1) [3], *Streptomyces albolongus* ATCC27414 (Csn21c, GenBank No. MG921183.1) [27], *Streptomyces* sp. N174 (SsCsn46, GenBank No. AAA19865.1) [16], *Bacillus amyloliquefaciens* ECU08 (BaCSN46B, GenBank No. MK257735.1) [28], *Bacillus subtilis* 168 (BsCsn, GenBank No. BSU_26890) [25], *Staphylococcus capitis* (Csn-CAP, GenBank No. WP_016898850.1) [29], *Bacillus coagulans* CK108 (TCH-2, GenBank No. AAL40906.1) [30], *Bacillus* sp. DAU101 (CSN-SP, GenBank No. ABC66094.1) [31], *Bacillus sp. strain* CK4 (choK, GenBank No. AAF24188.1) [32], *Burkholderia gladioli* CHB101 (BgcsnA, GenBank No. BAA82154.1) [33], *Nocardioides* sp. N106 (csnN106, GenBank No. AAA63405.1) [34], *Pseudomonas* sp. A-01 (Cto1, GenBank No. BAC06189.1) [35], *Streptomyces coelicolor* A3(2) (ScCsnA, GenBank No. CAB61194.1) [36], *uncultured bacterium* (CsnA, GenBank No. AJA30433.1) [37], *Gynuella sunshinyii* YC6258 (GsCsn46A, GenBank No. AJQ97965.1) [10], *Paenibacillus dendritiformis* (Csn-PD, GenBank No. WP_006679998.1) [38], *Bacillus* sp. BY01 (BsCsnB, GenBank No. MN531545.1) [39], *Bacillus* sp. Q1098 (BsCsnQ, GenBank No. MN963773.1) [40], *Janthinobacterium* sp. 4239 (Cho4239-1, GenBank No. ADB89905.1) [41], and *Streptomyces* sp. SirexAA-E (SACTE_5457, GenBank No. AEN13266.1) [24].

### 3.4. Homology Modelling of Sn1-CSN and Molecular Docking

Modelling was performed by I-TASSER [45,46], and crystal structures of two chitonases were used as templates, including those from *Streptomyces* sp. SirexAA-E (PDB ID: 4ILY) (https://www.rcsb.org/structure/4ily, accessed on 19 May 2021) [24] and *Streptomyces* N174 (PDB ID: 1CHK) [46].

To insight into the possible mode of action of Sn1-CSN with chitosan, its binding interactions with chitobiose and chitotriose were mimicked with the Surflex-Dock method in SYBYL-X software, respectively (Tripos Inc., St. Louis, MO, USA). For molecular docking, the hydrogen atoms were added, and the atomic charges were calculated with the MMFF94 method for protein and the Gasteiger–Huckel method for chitobiose and chitotriose. The binding sites of chitosanase with chitobiose and chitotriose were found with Automatic mode in Protomol generation, in which the Threshold and Bloat were set as 0.5 and 10 Å, respectively. The default values were used for all other parameters. To reduce the impact of initial ligand conformation on the docking results, three starting conformations of each ligand, which were obtained according to energy minimization with Max Iterations set to 0, 100, and 1000 were used. The highest TotalScore of each ligand was used to compare their binding interaction, and its conformation was used for structural analysis.

### 3.5. DNA Manipulation

Molecular cloning was done by following the standard protocol [47].The plasmid for the synthesized chitosanase gene cloned into the vector PUC57 at the restriction sites of *BamH*I (5′-terminal) and *Xho*I (3′-terminal) was digested with BamHI and XhoI and re-cloned into the vector pET-22b(+) digested with the same restriction enzymes. The final construct pET-22b-Sn1-CSN was confirmed by DNA sequencing.

### 3.6. Protein Over-Expression and Purification

The expression construct pET-22b-Sn1-CSN was overexpressed in *E. coli* BL21(DE3) in the presence of isopropyl-β-D-thiogalactopyranoside (IPTG) [47]. A single colony was cultured in 50 mL LB containing ampicillin (100 μg/mL) at 220 rpm and 37 °C overnight. The overnight culture was used to inoculate 100 mL LB medium with 100 μg/mL ampicillin. When OD_600nm_ was about 0.6, protein expression was induced at 16 °C with different IPTG concentrations and induction time. Finally protein was expressed under optimized conditions (0.1 mM IPTG, 16 °C for 12 h). The cells were harvested by centrifugation at 3950 *g* and 4 °C for 20 min.

All purification procedures were carried out at 4 °C. The cell pellet was suspended in 20 mL buffer A (50 mM Tris-HCl, pH 8.0, 0.5M NaCl), and 1 mM phenylmethanesulfonylfluoride (PMSF) was used as the protease inhibitor. The cell suspension was homogenized by sonication at 50% amplitude (working time 6 min). The crude protein solution was obtained by spinning down at 12,000 *g* and 4 °C for 20 min.

Nickel chelating resin (5 mL) was equilibrated with buffer A. The crude protein was loaded onto the column, which was washed with buffer A containing 20–500 mM imidazole in sequence. The enzyme purity was checked by SDS-PAGE. The fractions containing Sn1-CSN were combined and dialyzed against buffer A. The protein concentration was determined by the Bradford method using bovine serum alumin as a standard [48].

### 3.7. Enzyme Assay

All enzymatic assays were performed in triplicate. Chitosanase activity was quantified by measuring the amount of released reducing sugars from chitosan through the 3,5-dinitrosalicylic acid (DNS) method [49]. D-Glucosamine was used as a standard. The assay mixture (1 mL) was composed of 0.5% (*w*/*v*) chitosan and enzyme solution in 50 mM Britton-Robinson buffer (pH 5.5), and enzymatic reactions were done at 37 °C for 15 min. One unit (U) of chitosanase activity was defined as the amount of chitosanase required to release 1 μmol of reducing sugar per min under standard assay conditions, and specific activity was defined as units mg^−1^ chitosanase.

### 3.8. Determination of Optimal pH and pH Stability

The optimal pH of Sn1-CSN was carried out in 50 mM Britton-Robinson buffer at 37 °C and pH values between 2.0 and 11.0, and all enzymatic assays under different conditions were incubated for 15 min. Specific activities were calculated as above.

The pH stability assay was evaluated by first preincubating Sn1-CSN in 50 mM Britton-Robinson buffer at different pH values (pH 4.0–11.0) at 4 °C for 1 h, 5 h, 24 h, and 120 h, respectively. The residual activities were analyzed immediately under standard conditions (optimal pH, 37 °C for 15 min). The original activity at optimal pH (6.0) was considered as 100%, and the percentage of the residual activity at different time points and pH values against the original one at optimal pH (6.0) was calculated.

### 3.9. Determination of Optimal Temperature and Thermal Stability

The optimal temperature was determined in 50 mM Britton-Robinson buffer (pH 6.0) between 30 and 60 °C, and all enzymatic reactions at different temperatures were incubated for 15 min.

To determine the thermal stability of Sn1-CSN, it was pre-incubated for different intervals (15 min–2 h) at pH 6.0, and 30 °C, 35 °C, 40 °C, 45 °C, 50 °C, and 55 °C, respectively. and. The residual activities were measured under standard conditions (optimal pH, 37 °C for 15 min) after all samples were chilled on ice for at least 15 min. The original activity at pH 6.0 considered as 100%, and the percentage of the residual activity at different time points and temperatures against the initial one was calculated.

### 3.10. Determination of Kinetic Parameters

Kinetic parameters were measured under initial rate conditions through non-linear regression analysis of the Michaelis–Menten equation [50,51]. The initial rates of Sn1-CSN were analyzed at 37 °C in the presence of chitosan ranging from 0.1 to 1.5% (*w*/*v*) in a 50 mM Britton-Robinson buffer (pH 6.0). The released reducing sugars were quantified as above after being incubated for 15 min.

### 3.11. Effects of Metal Ions on Enzyme Activity

The impact of some metal ions on the catalytic activity of Sn1-CSN was investigated in the presence of 1 mM of various metal ions (Pb(CH_3_COO)_2_, NiSO_4_, CuSO_4_, BaCl_2_, ZnSO_4_, CoCl_2_, CaCl_2_, MgCl_2_, Fe_2_(SO)_3_, and Li_2_SO_4_). Since phosphate in Britton-Robinson buffer might affect the assay, it was replaced with 100 mM sodium acetate (pH 6.0). The reaction in the absence of metal ions was used as the control, which was taken as 100%. The percentage of the activity in the presence of different metal ions against the control was determined.

### 3.12. Hydrolysis of Chitosan by Sn1-CSN and Characterization

Hydrolysis of chitosan (DDA > 94%) (*w*/*v*, 0.5%) by Sn1-CSN was done in 1 mL of 1% acetic acid at 37 °C for 0.5 h, 1 h, 2 h, 3 h, and 24 h, respectively, and a reaction in the absence of Sn1-CSN was used as the control. The degradation products were analyzed by TLC and HPLC. For TLC analysis, samples were spotted on the silica gel plate, which was developed usingbutan-1-ol: acetic acid:water = 9:4:7) (*v*/*v*/*v*. Finally products were visualized by heating at 105 °C for 5 min after the plates were sprayed with 10% sulfuric acid in ethanol. HPLC analysis was carried out at Acchrom S6000 HPLC system (Acchrom Technologie, Dalian, China) with an Acchrom XAmide column (4.6 mm × 250 mm, 5 μm, Acchrom Technologie, Dalian, China). The used HPLC conditions were water (A) 0–25%, acetonitrile (B) 75–50% and ammonium formate (100 mM, pH 3.2) (C) 25% during 0-70 min. The flow rate was 1.0 mL/min, and the column temperature was 40 °C. The evaporation light-scattering detector (ELSD) was set at a probe temperature of Nev 55 °C and Eva 85 °C, and the nebulizer gas (nitrogen) was adjusted to 26 psi.

The hydrolysis products were also characterized by MALDI-TOFMS. MALDI-TOFMS spectra were recorded with a MALDI-TOF/TOF mass spectrometer (Bruker Ultraflextreme, Germany) with a nitrogen laser (337 nm), reflector and LIFT modes of operation. Instrument settings for the positive-ion mode: accelerating voltage, 25 kV; laser power, 30%; the number of shots, 100; laser shot rate, 66 Hz. Instrument settings for the negative-ion mode: accelerating voltage, −25 kV; laser power, 17%; the number of shots, 100; laser shot rate, 66 Hz. Sample preparation: a mixture containing 1 μL of the sample (0.1 mg/mL) and 1 μL of 0.5 M 2,5-dihydroxybenzoic acid (DHB) matrix solution in MeOH was introduced onto the sample plate and air-dried.

### 3.13. Hydrolysis of Partially Acetylated Chitosan by Sn1-CSN and Characterization

Partially acetylated chitosan was prepared according to the published procedure [52], and DDA was determined by ^1^H NMR following the standard procedure [53]. The hydrolysis procedure of partially acetylated chitosan and characterization of degradation products was carried out as above.

## 4. Conclusions

In summary, multiple sequence alignment and phylogenetic analysis of Sn1-CSN from *Streptomyces niveus* and homologous GH 46 *endo*-chitosanases suggested that Sn1-CSN is a new GH 46 *endo*-chitosanase. Sn1-CSN was overexpressed in *E. coli* and characterized in detail. It showed the highest activity at pH 6.0 and 50 °C. It was very stable between pH 4.0 and pH 11.0. Sn1-CSN exhibited high stability at ≤45 °C and was unstable above 50 °C. The apparent *K_m_* and *k_cat_* values of Sn1-Csn were 1.8 mg/mL and 88.3 s^−1^, respectively. Cu^2+^ showed some stimulatory effects on Sn1-CSN, while Sn1-CSN was inhibited by Fe^3+^. The structural characteristics of GH 46 *endo*-chitosanases were demonstrated by structure modelling of Sn1-CSN and molecular docking. The main hydrolysis product of chitosan (degree of deacetylation, DDA > 94) by Sn1-CSN was chitobiose (87.3%), whereas degradation products of partially acetylated chitosan (DDA 69%) were very complicated with DP 2-15. This *endo*-chitosanase could be potentially for the production of chitobiose and partially acetylated COS.

## Data Availability

The data presented in this study are available in Supplementary Materials associated with this article.

## Supplementary Materials

The following are available online at https://www.mdpi.com/article/10.3390/md19060300/s1, The codon-optimized gene sequence of *Sn1-CSN.* Figure S1. Structure modelling of Sn1-CSN by I-TASSER. Figure S2. SDS-PAGE analysis of purified Sn1-CSN. Table S1. Comparison of enzymological properties of some characterized GH 46 *endo*-chitonases. Figure S3. Thin-layer chromatography (TLC) analysis of hydrolysis products of partially acetylated catalyzed by Sn1-CSN over time. Table S2. Comparison of effects of metal ions on some characterized GH 46 *endo*-chitonases.
