# Supplementary material for: Expression and Biochemical Characterization of a Novel Marine Chitosanase from Streptomyces niveus Suitable for Preparation of Chitobiose
Source: Mar Drugs. 2021 May 24;19(6):300. doi: 10.3390/md19060300 (PMC8225178; doi:10.3390/md19060300)
Supplement: Supplementary file 1 [file marinedrugs-19-00300-s001.zip › marinedrugs-1199767-supplementary.pdf]

Supplementary Materials

Expression and biochemical characterization of a novel marine chitosanase from *Streptomyces niveus* suitable for preparation of chitobiose

Tong Chen[1,2], Gong Cheng[2], Siming Jiao[2], Lishi Ren[2], Chuanfang Zhao[3], Jinhua Wei[2], Juntian Han[2], Meishan Pei[1,*], Yuguang Du[2,*], Jian-Jun Li[2,*]

[1]School of Chemistry and Chemical Engineering, University of Jinan, Jinan 250022, China.

[2]National Key Laboratory of Biochemical Engineering, National Engineering Research Center for Biotechnology (Beijing), Key Laboratory of Biopharmaceutical Production & Formulation Engineering, PLA, Institute of Process Engineering, Chinese Academy of Sciences, Beijing 100190, China.

[3]State Key Laboratory of Environmental Chemistry and Eco-toxicology, Research Center for Eco-Environmental Sciences, Chinese Academy of Sciences, Beijing 100085.

*Corresponding authors: Meishan Pei, Phone: +86 531 89736800, Fax: +86 531 89736800, E-mail: chm_peims@ujn.edu.cn; Yuguang Du, Phone: +86 10 8254 5070, Fax: +86 10 8254 5039, E-mail: ygdu@ipe.ac.cn; Jian-Jun Li, Phone: +86 10 8254 5039, Fax: +86 10 8254 5039, E-mail: jjli@ipe.ac.cn.

Contents

1. The codon-optimized gene sequence of *Sn1-CSN*
2. **Figure S1.** Structure modelling of Sn1-CSN by I-TASSER
3. **Figure S2.** SDS-PAGE analysis of purified Sn1-CSN
4. **Table S1**. Comparison of enzymological properties of some characterized GH 46 *endo*-chitonases. ND, not determined in the references.
5. **Figure S3.** Thin-layer chromatography (TLC) analysis of hydrolysis products of partially acetylated catalyzed by Sn1-CSN over time
6. **Table S2.** Comparison of effects of metal ions on some characterized GH 46 *endo*-chitonases. ND, not determined

The codon-optimized gene sequence of *Sn1-CSN*

ATGGTTGCTCATGCTGCTCCAGGTGCTGATAGAGCTGGTGCTACTGCTGCT
GTTGTTGCTGCTCCTGGTTTGGATGATCCAGCCAAGAAAGAAATCGCTATG
CAGTTGGTTTCCTCCGCTGAGAACTCTTCATTGGACTGGCAAGGTCAGTAC
GGTTACGTTGAGGATATTGGTGACGGTAGAGGTTACACTGCTGGTATCATTG
GTTTCTGCTCTGGTACTGGTGACATGTTGGACTTGGTTGAGTTGTACACTG
AGAGAGAGCCAGACAACCCTTTGGCTTCTTACTTGCCAGCTTTGAGAGCTG
TTGACGGTACTGATTCTCACGAAGGTTTGGACCCAGGTTACCCAGATGCTT
GGAGAGAAGCTGCTGCTGATCCAGCTTTTAGAACTGCTCAAAACGACGAG
AGAGACAGGGTCTACTTTAACCCAGCTGTCTCCAGAGGTAAAGAGGATGG
CGTTGGTACTTTGGGTCAGTTCATCTACTACGACGCCATCGTTATGCACGGT
GATGGTAACTCTGACACCTCATTCTCCGGTATCAGAGAGAGGGCTTTGAAC
CAGGCTAGACCACCATCTGAAGGTGGTGACGAAACTGCTTACTTGAACGC
TTTCTTGGACGCTAGAGTTTGGGCCATGAAGCAAGAAGAAGCTCACGAGG
ACACTTCCAGAGTTGATACTGCTCAGAGAGTTTTCCTGGACGCCGGTAACT
TGAATTTGGACCCTCCATTGGATTGGAAGGTTTACGGTGAGGACTTCCACA
TTGGATAA

Figure S1. Structure modelling of Sn1-CSN by I-TASSER. Crystal structures of two chitonases from *Streptomyces* sp. SirexAA-E (PDB ID: 4ILY) and *Streptomyces* N174 (PDB ID: 1CHK) were used as threading templates for homology model of Sn1-CSN.

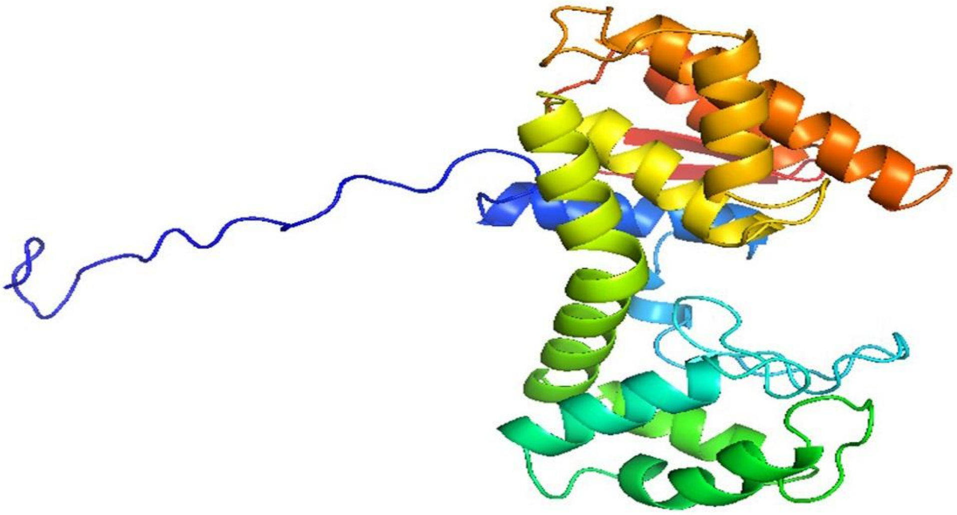

Figure S2. SDS-PAGE analysis of purified Sn1-CSN

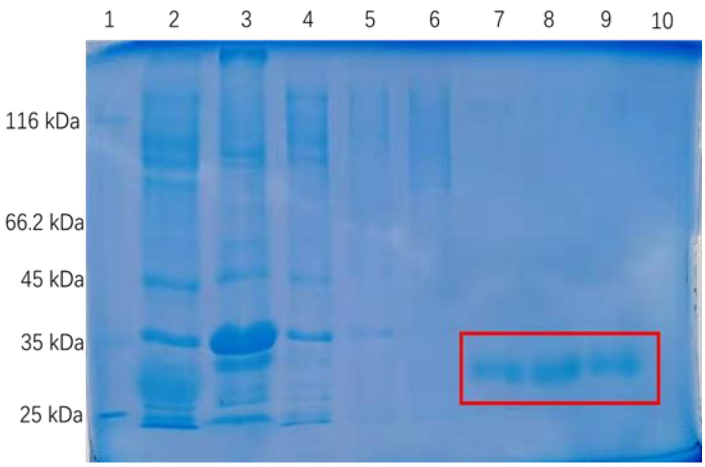

Lane1: molecular marker; Lane 2: crude protein; Lane 3: precipitate; Lane 4: flow through when loaded; Lane 5: eluents of buffer A (50 mM Tris/HCl, pH 7.9, 0.5 M NaCl); Lane 6: eluents of buffer A + 20 mM imidazole; Lanes 7-8: eluents of buffer A +50 mM imidazole; Lane 9: eluents of buffer A +160 mM imidazole; Lane 10: eluents of buffer A + 500 mM imidazole.

Table S1. Comparison of enzymological properties of some characterized GH 46 endo-chitonases. ND, not determined.

Name	Source	Optimal pH	pH stability	Optimal temperature (°C)	Thermal stability (°C)	K_m (mg/ml)	V_{max} (µmol/min/mg)	Products (DP)	Reference
Sn1-CSN	*Streptomyces niveus*	6.0	4.0-11.0	50	≤ 45	1.8	1800	2-4	This study
BaCsn46A	*Bacillus amyloliquefaciens* YX01	6.0	5.0-9.5	50	≤ 50	2.8	7142.9	2-10	[14]
Csn-BAC	*Bacillus sp.* MD-5	7.0	5.5-8.5	40	≤ 50	ND	ND	2,3	[3]
Csn21c	*Streptomyces albolongus* ATCC 27414	8.0	6.0-10.0	50	≤ 65	7.4	263.1	1,2	[27]
SsCsn46	*Streptomyces* sp. N174	5.5	5.0-6.5	65	≤ 45	0.029	27	3-5	[16]
BaCSN46B	*Bacillus amyloliquefaciens* ECU08	6.5	3.0-10.5	55	≤ 55	ND	ND	2,3	[28]
BsCsn	*Bacillus subtilis* 168	5.0-6.0	2.0-9.0	40-50	0-45	1.57	31800	2-6	[25]
Csn-CAP	*Staphylococcus capitis*	7.0	5.0-7.0	30	≤ 50	ND	ND	2,3	[29]
TCH-2	*Bacillus coagulans* CK108	6.5	ND	65	≤ 80	1.7 mM	516	1-6	[30]
CSN-SP	*Bacillus* sp. DAU101	7.5	ND	50	ND	ND	ND	2-4	[31]

choK	*Bacillus* sp. strain CK4	7.5	ND	55	≤ 80	0.8	173	2-6	[32]
BgcsnA	*Burkholderia gladioli* CHB101	5.6	ND	55	ND	ND	ND	2-4	[33]
ScCsnA	*Streptomyces coelicolor* A3(2)	4.1	ND	50	ND	0.054	37	ND	[36]
CsnA	uncultured bacterium	6.0	4.5-6.5	55	≤ 55	7.29	2373	2-6	[37]
GsCsn46A	*Gynuella sunshinyii* YC6258	5.5	4.0-9.0	30	≤ 40	1.97	385.6	2-4	[10]
Csn-PD	*Paenibacillus dendritiformis*	7.0	6.0-7.0	45	≤ 50	ND	ND	2	[38]
BsCsnB	*Bacillus* sp. BY01	5.0	3.0-7.5	35	< 30	ND	ND	2,3	[39]
BsCsnQ	*Bacillus* sp. Q1098	5.31	3.6-9.8	60	≤ 30	ND	ND	2,3	[40]
Cho4239-1	*Janthinobacterium* sp. 4239	5.0	ND	45	≤ 50	ND	ND	2,3	[41]
SACTE_5457	*Streptomyces* sp. SirexAA-E	6.0	ND	35-55	ND	2.2	25	2,3	[24]
csnN106	*Nocardioides* sp. N106	ND	ND	ND	ND	ND	ND	2,3	[34]
Cto1	*Pseudomonas* sp. A-01	5.0	5.0-8.0	ND	ND	ND	ND	ND	[35]

Figure S3. Thin-layer chromatography (TLC) analysis of hydrolysis products of partially acetylated catalyzed by Sn1-CSN over time. Lane 1, standard chitosan oligomers (DP 2-6); Lane 2, glucosamine (GlcN); Lanes 3-7, enzymatic hydrolysates of partially acetylated chitosan after 0.5 hr, 1 hr, 2 hr, 4 hr and 24 hr. The mixture (butan-1-ol : acetic acid : water = 9:4:7) (v/v/v) was used as the mobile phase to develop TLC plate.

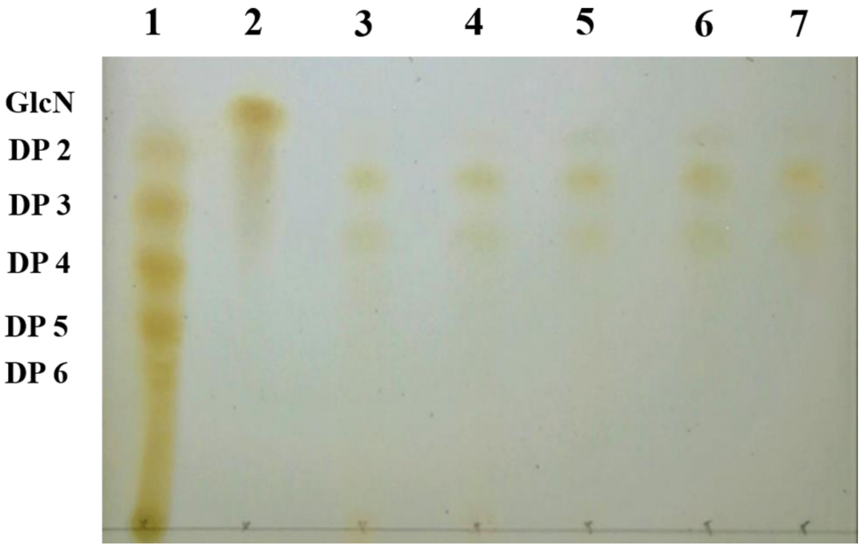

Table S2. Comparison of effects of metal ions on some characterized GH 46 *endo*-chitonases. ND, not determined in the references.

	Ni^{2+}	Ba^{2+}	Ca^{2+}	Cu^{2+}	Pb^{2+}	Zn^{2+}	Co^{2+}	Mg^{2+}	Fe^{3+}	Li^{+}	Ref
Sn1-CSN	No effect	No effect	No effect	+ 54.2%	No effect	+ 6.9%	No effect	No effect	- 20%	No effect	This study
Csn-BAC	- 85%	No effect	+ 7%	+ 168%	ND	- 9%	+ 103%	No effect	ND	ND	[3]
Csn21c	ND	No effect	- 4.6%	+ 3.9	ND	- 14.5%	No effect	- 11.9%	- 66.1%	ND	[25]
Csn-CAP	- 22%	- 4%	- 19%	+ 38%	ND	+ 10%	+ 4%	- 10%	ND	ND	[27]
CSN-SP	- 97%	- 34%	+ 31%	- 83%	ND	- 54%	- 53%	+ 3%	ND	+ 15%	[29]
Cto1	- 84%	- 5%	- 9%	- 100%	No effect	- 12%	- 86%	No effect	- 9%	No effect	[33]
CsnA	- 99.4%	- 37.8%	- 33.1%	- 99.4%	- 99%	- 98.7%	- 71%	- 46.2%	- 99.7%	- 37.3%	[35]
BsCsnB	ND	+ 11%	+ 9%	+ 36%	ND	ND	- 11%	+ 32%	- 96%	+ 30%	[37]
BsCsnQ	ND	+ 9%	+ 12%	+ 8%	ND	+ 14%	+ 10%	+ 6%	- 12%	+ 6%	[38]
